# Single‐vesicle imaging and co‐localization analysis for tetraspanin profiling of individual extracellular vesicles

**DOI:** 10.1002/jev2.12047

**Published:** 2021-01-11

**Authors:** Chungmin Han, Hyejin Kang, Johan Yi, Minsu Kang, Hyunjin Lee, Yongmin Kwon, Jaehun Jung, Jingeol Lee, Jaesung Park

**Affiliations:** ^1^ Department of Mechanical Engineering Pohang University of Science and Technology Pohang Gyeong‐buk Republic of Korea; ^2^ School of Interdisciplinary Bioscience and Bioengineering Pohang University of Science and Technology Pohang Gyeong‐buk Republic of Korea

**Keywords:** density gradient ultracentrifugation, EV heterogeneity, EV subpopulations, single‐vesicle analysis, size exclusion chromatography, tetraspanin markers

## Abstract

Extracellular vesicles (EVs) are secreted nano‐sized vesicles that contain cellular proteins, lipids, and nucleic acids. Although EVs are expected to be biologically diverse, current analyses cannot adequately characterize this diversity because most are ensemble methods that inevitably average out information from diverse EVs. Here we describe a single vesicle analysis, which directly visualizes marker expressions of individual EVs using a total internal‐reflection microscopy and analyzes their co‐localization to investigate EV subpopulations. The single‐vesicle imaging and co‐localization analysis successfully illustrated the diversity of EVs and revealed distinct patterns of tetraspanin expressions. Application of the analysis demonstrated similarities and dissimilarities between the EV fractions that had been acquired from different conventional EV isolation methods. The analysis method developed in this study will provide a new and reliable tool for investigating characteristics of single EVs, and the findings of the analysis might increase understanding of the characteristics of EVs.

## 1 INTRODUCTION

Extracellular vesicles (EVs) are cell‐secreted nano‐sized vesicles that have important functions in diverse biological activities (Février & Raposo, 2004; Raposo & Stoorvogel, 2013). EVs are complex bodies composed of various biological materials, including proteins, lipids, and nucleic acids (Théry et al., 2002). Traditional omics approaches have identified hundreds of proteins, nucleic acids, and lipids of EVs (Choi et al., 2015; Guduric‐Fuchs et al., 2012; Ji et al., 2014; Llorente et al., 2013; Simpson et al., 2008; Skotland et al., 2019). Due to the wide range of components, cells can produce various types of EVs. Consequently, EVs are expected to deliver more comprehensive and specific messages than soluble molecules (Kao & Papoutsakis, 2019; Krämer‐Albers & Hill, 2016). However, most conventional analytical methods for EVs consider ensemble averages of heterogeneous populations, so much remains unknown about the heterogeneity of EVs. The ensemble methods such as western blotting (WB) and omics approaches require lysis of EVs, and only provide pooled information about the whole population, so information from individual EVs is lost (Margolis & Sadovsky, 2019). As a result, despite the vast amount of existing information about EV components, the characteristics of individual EVs and heterogeneity of EV samples cannot be discerned.

Some studies have attempted to elucidate the heterogeneity of EVs, and have suggested the presence of different EV subpopulations depending on isolation methods (Shu et al., 2020). Investigations using density gradient ultracentrifugation (DG) and size exclusion chromatography (SEC) has revealed the presence of EV populations having different densities and sizes (Böing et al., 2014; Kowal et al., 2016). Studies that exploit asymmetric‐flow field‐flow fractionation suggested distinct EV subpopulations having different sizes ranging from approximately 35 to 120 nm that also have different biological compositions (Sitar et al., 2015; Zhang et al., 2018). Although such isolation‐oriented approaches have successfully separated EV populations into relatively smaller populations, the implications of these studies were mostly limited to the evaluation of physical characteristics (Gardiner et al., 2013; Höög & Lötvall, 2015; Yuana et al., 2013). Therefore, to better understand the correlation between EV subpopulations and biological implications, biological properties and heterogeneity of refined EV populations should be further investigated.

A few advanced analytical methods have also developed for characterizing biological properties of individual EVs. High resolution flow cytometers and imaging flow cytometers have been proposed to overcome the limitations of conventional cytometers that are optimized for single‐cell analysis, but they still have technical limitations such as swarm detection and low detection sensitivity (detection limits are often > 200 nm) (Erdbrügger et al., 2014; Van Der Pol et al., 2012). NTAs equipped with fluorescence units were developed for probing biological properties of individual EVs, but the technical principle exploits diffusion, and therefore cannot reliably investigate the co‐expression of multiple markers. Recently, studies have used advanced fluorescence microscopies such as super resolution microscopies and con‐focal microscopy to analyse multiple protein expressions of individual EVs (Chen et al., 2016; Lee et al., 2018; Nizamudeen et al., 2018). In addition, technologies that do not exploit fluorescence have also been proposed to investigate individual EVs; examples include single‐particle interferometric reflectance imaging sensing (SP‐IRIS), nano‐plasmonic sensors and Raman spectroscopic analysis (Daaboul et al., 2016; Im et al., 2014; Lee et al., 2018). Although such single‐EV analysis approaches have provided important findings regarding EV heterogeneity and subtypes, this field of study still needs a method that can precisely analyse multiple biological markers of individual EVs without the bias that may arise during sample preparation and analyses.

Recently, technological advances in biophysical methods have enabled observation of the behaviour of single biological molecules (Aggarwal & Ha, 2016; Roy et al., 2008). Applications of single‐molecule imaging techniques have been used to characterize interactions between biological molecules, and have revealed many phenomena that had been obscured by ensemble‐averaging methods (Jain et al., 2011; Myong et al., 2006). Particularly, single‐molecule pull‐down (SiMPull) and single‐molecule blotting (SiMBlot) assays overcome the limitation of conventional ensemble averaging biochemical analysis such as WB, which have enabled us to quantitatively characterize biochemical properties of individual biomolecules (Jain et al., 2011; Kim et al., 2016). The techniques have also been applied to relatively large biological complexes, such as viruses and liposomes, but they have rarely been used to explore the characteristics of EVs (Brandenburg & Zhuang, 2007; Choi et al., 2010).

In this study, to overcome the technical pitfalls of conventional EV analyses, we developed a single‐vesicle imaging analysis method that uses total internal reflection fluorescence microscopy (TIRFM). The analysis can visualize multiple marker expressions of individual EVs by using fluorescent probes, and can also investigate EV subpopulations by analysing co‐localization of markers. We used the single‐EV tetraspanin co‐localization analysis to investigate EV fractions that were isolated by three frequently‐used EV isolation methods. The analysis revealed that individual EVs had distinct tetraspanin expression patterns that could not be characterized by conventional analyses, and this capability enabled us to deduce similarities and dissimilarities among the conventional methods to isolate EVs.

## 2 METHODS

### 2.1 Cell culture

The HEK293 WT (# 21573), MCF‐7 (# 30022) and B16BL6 (# 80006) cell line was purchased from Korean Cell Line Bank (KCLB). The HEK293 WT cells were maintained in Dulbecco's modified Eagle's medium (DMEM, Gibco, 12100046) supplemented 10% (v/v) fetal bovine serum (FBS, Gibco, 12483020) and 1x antibiotic‐antimycotic (anti‐anti, Gibco, 15240062) at 37°C and 5% CO_2_ in a humidified incubator. MCF‐7 and B16BL6 cells were maintained in minimum essential medium (MEM, Gibco, 41500‐034) supplemented 10% (v/v) FBS and 1x anti‐anti at 37°C and 5% CO_2_ in a humidified incubator. When cells were ∼ 90% confluent, culture media were changed to supplement‐free base medium and cultured for 24 h under the same incubation condition. After 24 h, cultured media were collected and then centrifuged at 500 × *g* for 10 min to remove detached cells and at 3000 × *g* for 20 min to eliminate cellular debris. The pre‐cleaned media (supernatants) were stored at −80°C until they were used. A total of 6 L of cell cultured media (CM) was pooled and processed for EV isolation to minimize batch to batch variation.

### 2.2 Antibodies and labelling reagents

A CD9 (MEM‐61, sc51575), CD63 (MX‐49.129.5, sc5275), CD81 (1.3.3.22, sc7637), Calnexin (H‐70, sc11397) and ribosomal protein S6 (C‐8, sc‐74459) primary antibodies and HRP‐conjugated anti‐mouse IgG_1_ secondary antibody (sc2005) were purchased from Santa Cruz Biotechnology for western blotting. Alexa‐Fluor 488 (A‐21121), 546 (A‐21123), and 647 (A‐21240) conjugated anti‐mouse IgG_1_ secondary antibodies were purchased from Invitrogen. Fluorescent dye‐conjugated primary antibodies for CD9, CD63, and CD81 (MEM‐61, MX‐49.129.5, and 1.3.3.22 clones, respectively) were purchased from Novus biologicals, BioLegends, and Santa Cruz Biotechnology, and lot to lot variations of labelling efficiency was tested before use. Specifically, the signal counts of conjugated antibodies were compared with the counts of same clones of primary antibodies and secondary antibodies, and the conjugated antibodies that yielded > 90% of the counts of indirect labelling were selected for further multi‐colour single‐vesicle analyses. A recombinant CTB protein (NBP2‐61449) and anti‐CTB rabbit polyclonal antibody (NB100‐63067) were purchased from Novus Biological. A Di‐dye cell labelling kit (V22889) and Alexa‐Fluor 488 conjugated annexin V (A13201) were purchased from Invitrogen.

### 2.3 EV preparation: concentration, biotinylation and purifications

For differential ultracentrifugation (DUC) concentration, Type 45 Ti (Beckman) fixed‐angle titanium rotor was used for first and second rounds of EV pelleting. The procedures of DUC concentration were derived from previous literature (Théry et al., 2006). The 6 L of pooled cultured media (CM) was centrifuged at 500 × *g* for 10 min to remove cells then centrifuged again at 3000 × *g* for 20 min to remove cellular debris. The pre‐cleaned CM was them ultra‐centrifuged at 100,000 × *g* for 2 h, and the resulting pellets were re‐suspended in total 12 ml filtered‐PBS solution. After first‐round pelleting, the sample was biotinylated with approximately 100‐times molar excess of sulfo‐NHS‐biotin (Thermo scientific, 21217) according to the manufacturer's instruction. The biotinylated sample was ultra‐centrifuged again at 100,000 × *g* for 2 h to remove protein contaminants and residual biotins. The pellet was suspended again in 4 ml filtered‐PBS and centrifuged again at 3000 × *g* for 20 min to remove EV aggregates formed during ultracentrifugation; 1 ml of the EV solution was kept for the characterization of DUC method (DUC‐EVs) and 3 ml of the solution was used for further purification. Each purification method was performed using 1 ml of DUC‐EVs. Because 1 ml of DUC samples was prepared from 1.5 L CM, each purification method can be considered to isolate EVs from initial 1.5 L CM. In addition, the DUC sample had already been biotinylated during the concentration process, so purification methods did not require a biotinylation process.

For density gradient ultracentrifugation (DG) and buoyant DG (BDG) purification, different densities of Opti‐Prep iodixanol density‐gradient medium (AXIS‐SHIELD) were prepared according to the manufacturer's instruction. The overall procedures of DG and BDG purifications were based on the previous literature with minor modifications (Hong et al., 2009; Tauro et al., 2012; Wubbolts et al., 2003). In the DG method, a sample is loaded on top of the density layers, thus the DUC sample was diluted with PBS (0%) and layered on top of 30%, 20% and 10% Opti‐Prep layers. On the contrary, in the BDG method, a sample is loaded at the bottom with the highest‐density layers, so the DUC sample was diluted in 30% Opti‐Prep layers and layered at the bottom of tube with 20% and 10% Opti‐Prep and PBS (0%) layers. The DG and BDG samples were centrifuged at 100,000 × *g* for 2 h using a SW55 Ti swinging‐bucket rotor (Beckman) with no‐brake option. All fractions between the layers (DG/0‐10, DG/10‐20, DG/20‐30, BDG/0‐10, BDG/10‐20 and BDG/20‐30) were collected and stored for further analyses.

We performed SEC purification as described previously (Böing et al., 2014). Approximately ∼7.5 ml bed volume of Sepharose CL‐2B (GE Healthcare) gel‐filtration matrix was packed into a 10‐ml plastic disposable column (Pierce). The packed columns were washed using more than three bed volumes (∼ 30 ml) of filtered‐PBS solution before use. A 1‐ml of DUC sample was loaded on the column and then eluted with filtered‐PBS solution. The eluates were collected in 20 fractions of 0.5 ml and stored for further analyses.

### 2.4 Conventional EV characterizations

All fractions of EVs were characterized by western blotting (WB, Bio‐Rad), nano‐particle tracking analysis (NTA, ExoCope, ExosomePlus), and transmission electron microscopy (TEM, JEOL) according to the guideline provided by international society for extracellular vesicles (ISEV) (Théry et al., 2018).

EV‐positive (CD9, CD63, CD81) and EV‐negative (Calnexin and ribosomal protein S6) markers were used for WB analysis. In WB analysis, the same volumes (25 μl) of samples were lysed and separated by SDS‐PAGE in a non‐reducing condition (Bio‐Rad). The separated proteins were transferred to a PVDF membrane, and blocked for 1 h at room temperature (RT) using a solution of 3% (w/v) BSA in TBS supplemented with 0.05% (w/v) Tween‐20. The membrane was then incubated with primary antibodies (200 ng/ml) in the blocking solution overnight at 4°C, and subsequently incubated with HRP‐conjugated secondary antibodies (200 ng/ml, Santa Cruz Biotechnology) for 1 h at RT. The membrane was exposed to a chemiluminescent substrate (ECL, Thermo Scientific) for detection. The signals were captured using a c‐Digit western blot scanner (LI‐COR). The WB results presented in the same figure set were all detected under the same condition for valid comparison. Quantification of WB bands was performed using ImageJ software. Briefly, a scan of WB gel was inversed and a fixed size of rectangular region of interest (ROI) that include each WB band was selected. The intensities of bands were measured as mean intensity of ROIs.

Particle concentrations and sizes of samples were measured using NTA (Exocope, Exosomeplus). All samples were diluted ∼1,000 to 3,000 times with filtered‐PBS solution to achieve appropriate particle concentration (∼100 particles/imaging field). Images of all samples were recorded for 15 s at least six times in different imaging fields. The size distribution of EVs was plotted using mean values of six measurements.

For TEM analysis, each EV fraction was loaded onto formvar carbon film (Electron Microscopy Science) for 30 min at RT. The sample‐loaded grids were then negatively stained with 2% uranyl acetate (Sigma) for ∼ 10 s for image contrasting. The grids were then completely dried overnight and imaged using a TEM (JEOL).

### 2.5 Surface preparation and EV immobilization

The detailed procedure used to prepare the DDS‐tween 20 surface was explained previously, and used with minor modification (Hua et al., 2014). Briefly, cover and slide glasses were extensively cleaned with acetone, methanol (Samchun Chemicals), and ultra‐pure water. The glasses were then incubated in piranha solution (three parts sulfuric acid; one part 30% H_2_O_2_) for surface cleaning and activation, and then rinsed thoroughly with ultra‐pure water. The activated glasses were incubated with DDS (Sigma) solution in cyclohexane for 1.5 h at RT, and then rinsed with clean cyclohexane and completely dried under N_2_ gas in a fume hood. The DDS‐treated glasses were then sealed with N_2_ gas and stored at −20°C for up to 2 weeks.

For EV immobilization, DDS‐treated glasses were first assembled in a simple flow chamber by using double‐sided tape (3 M) and epoxy bond (Devcon). After the seal of each chamber had completely cured, it was incubated with 0.2 mg/ml biotin‐BSA (Sigma) for 5 min at RT to introduce biotin sites to the surface, then incubated with 0.2% Tween‐20 (Sigma) solution for 10 min at RT to passivate the remaining surface. To prepare a control surface without biotin anchor, DDS‐treated glasses were directly passivated with 0.2% Tween‐20 solution. BSA and Tween‐20 solutions were both prepared in tris buffer (50 mM Tris). The passivated chambers were incubated with 0.4 mg/ml NeutrAvidin (Thermo Scientific) solution for 5 min at RT, and then biotinylated EVs were introduced to the chambers and incubated for 10 min at RT. NeutrAvidin and EV samples were prepared with filtered‐PBS supplemented with 0.5 mg/ml BSA. For surface‐tethered CD81 antibody immobilization of EVs, the surface was prepared in the same way with DDS‐Tween surface up to the avidin incubation step. Then, biotinylated‐CD81 antibodies were incubated for 10 min, then EV samples were introduced to the chamber and incubated for 10 min at RT for immobilization.

### 2.6 Scanning probe microscopy and scanning electron microscopy

Surfaces with immobilized‐EVs were fixed with 3% (v/v) electron microscopy grade glutaraldehyde (Sigma) for 15 min at RT. Surfaces were then washed with PBS at least three times. Fixed samples were stained with 1% (w/v) osmium tetroxide (OsO4, Sigma) for 30 min at RT, and the OsO_4_ was removed by thorough washing with distilled water (DW) at least three times. Samples were then incubated with 1% carbohydrazide (Sigma) for 20 min at RT, and washed with DW at least three times. Then, samples were dehydrated by sequential immersion in 20%, 40%, 60%, 80%, and 100% ethanol solution (v/v) over 30 min at RT. Dehydrated samples were completely dried using a critical point dryer for at least 30 min. For the SPM analysis, the dehydrated surface analyzed using an AFM (Veeco Dimension 3100, VEECO) for an area of 5 × 5 μm with tapping mode in 512 lines at a scan rate of ∼0.7 Hz (Skliar & Chernyshev, 2019). An AppNano Si probe (ACT‐50, AppNano) was used for the analysis (rectangular‐shaped cantilever with nominal length = 125 μm and width = 30 μm; and a pyramidal‐shaped tip with height 14–16 μm, and tip radius < 10 nm, spring constant 13–77 N/m and *f* = 300 kHz). For SEM analysis, the sample was further sputter‐coated with platinum (20 mA, 10 s) and analyzed using a JSM7401F high resolution FE‐SEM (JEOL).

### 2.7 Individual EV visualization and co‐localization analysis

EV‐immobilized surfaces were fluorescently visualized using antibodies. For indirect antibody labelling, 2 μg/ml (∼13.3 nM) primary antibodies were first introduced to the chamber for 10 min at RT, and then 1 μg/ml (∼6.6 nM) fluorescent conjugated secondary antibodies were introduced for 8 min at RT. When conjugated antibodies were used, 5 μg/ml (∼33.2 nM) conjugated antibodies were incubated for 10 min at RT. When multiple conjugated antibodies were used for labelling, all antibodies were mixed together, and then used for EV labelling (cocktail staining). After the labelling, the chambers were thoroughly washed with filtered PBS three times. The fluorescent labelled EVs were visualized using a laboratory‐built objective‐type TIRF microscope (Olympus, IX73) equipped with an EMCCD (Andor, iXon 888), four diode lasers (Cobolt, 405‐nm, 488‐nm, 638‐nm MDL series, and 561‐nm DPL series), and a four‐channel simultaneous‐imaging system (Photometrics, QV2). Average signal count per field was measured from 2,000 μm (Février & Raposo, 2004) imaging areas.

For co‐localization analysis, we built image processing software in MATLAB by reference to previous literature (Jain et al., 2011; Kim et al., 2016; Ulbrich & Isacoff, 2008). Briefly, the coordination of each multi‐channel images was precisely corrected using a MATLAB‐coded image processing program. The coordinates were corrected using the coordinates of the signals acquired from the full‐range fluorescent bead for calibration (Spherotech, FP‐0257‐2). Then the centers of each tetraspanin signals of coordinate‐corrected images were determined with sub‐pixel accuracy using Gaussian fitting methods. Using the acquired center coordinates of each signals, the distances between the signals were calculated, and the signals located within 3 pixels (∼300 nm) were considered to be co‐localized.

### 2.8 Multi‐dimensional analysis

Populations of samples were compared using two different multi‐dimensional analyses: t‐stochastic neighbour embedding (t‐SNE) and a clustered heat map based on Euclidean distances. In t‐SNE, nine independent population data of 11 different fractions were plotted as a single point in two‐dimensional space with different perplexity values, and the value that best illustrated the clusters and that had low error was selected. The analysis was performed using a computer code derived from the tsne library of the R package. In clustered heat map analysis, Euclidean distances between the 11 different population data were calculated, and the clusters were formed based on the similarity and dissimilarity values, which were calculated using a computer code derived from ggplot2 and pheatmap libraries of the R package (‘pheatmap’ and ‘tsne’) (Donaldson, 2016; Kolde, 2019; R Core Team 2020)

### 2.9 Statistical analysis

Data visualization were performed using Excel (Microsoft) and packages in R statistics software. The data were presented as mean ± SD. When statistical analysis was required, two‐tailed unpaired Student's t‐test was performed. Pairs that had *P* < 0.05 and *P* < 0.01 were considered statistically significant, and noted using a single asterisk and double asterisk, respectively.

## 3 RESULTS

### 3.1 Schematic of single vesicle imaging and co‐localization analysis

**FIGURE 1:**
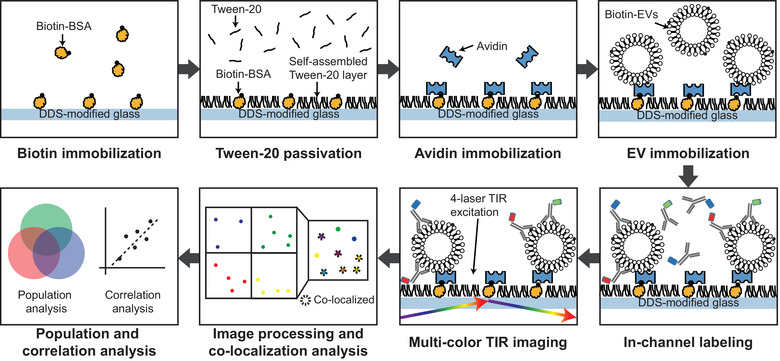
Procedure of single‐vesicle imaging and co‐localization analysis. A simple fluidic channel made of DDS functionalized cover and slide glasses was incubated with biotinylated BSA, then passivated with Tween‐20. Avidins were then introduced to the surface to immobilize biotinylated EVs. Unbound molecules were washed out after each step to prevent unwanted interactions among the molecules. Immobilized EVs were labelled with probes, and the EVs were imaged with multiple excitation lasers and a multi‐colour simultaneous fluorescence imaging device equipped with EMCCD camera. Acquired signals were analyzed for signal counts and co‐localizations for investigating EV heterogeneity and subpopulation

The analysis method (Figure 1) visualizes individual EVs and analyzes their co‐localization by immobilizing them to a surface that can stably anchor EVs and effectively repel non‐specific adsorptions of probes. To achieve such characteristics, we utilized a glass substrate functionalized with dichlorodimethylsilane (DDS) and Tween‐20 passivation that had been developed for single‐molecule imaging (Hua et al., 2014). Due to the characteristics of the imaging surface, EVs can be efficiently labelled with fluorescent probes within 20 min. The expressions of multiple markers on individual EVs were detected using a multi‐colour fluorescence imaging system that consists of multiple TIR‐aligned excitation lasers and an electron‐multiplying CCD (EMCCD) camera. The co‐localizations of detected signals were analyzed with sub‐pixel accuracy by using an computer‐coded image processing program described in previous studies (Kim et al., 2016). The 0.2% Tween‐20 solution only acted as passivation layer; redundant Tween‐20 was thoroughly washed out using sample buffer (PBS). According to the previous literature both the microvesicles (MVs) and exosomes are not lysed even when 5% Tween‐20 is added (Osteikoetxea et al., 2015). Therefore, the use of Tween‐20 does not disrupt the structure or characteristics of EVs and also does not compromise antibody activities.

### 3.2 Validation of single vesicle imaging surface

**FIGURE 2:**
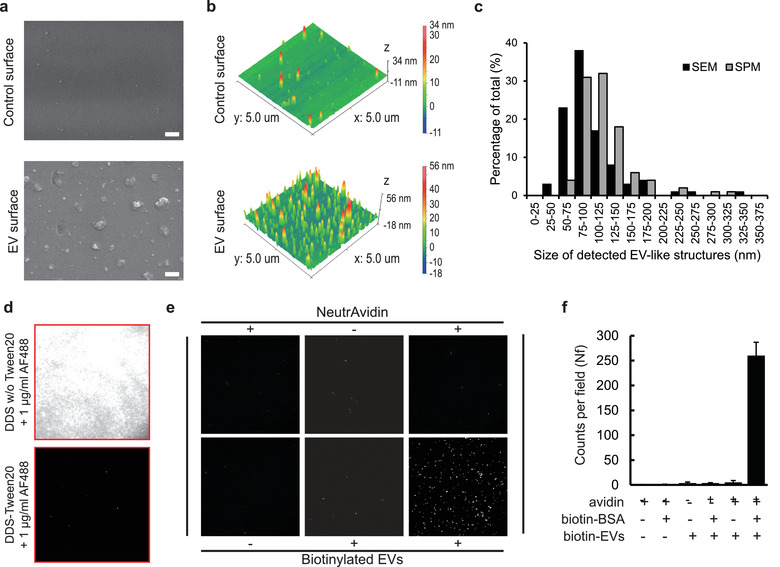
Validation of single‐EV imaging surface. (a) Scanning electron microscopy (SEM) images of control surface (top) and EV immobilized surface (bottom). Only the EV immobilized surface showed vesicle‐like structures, x 50k magnification, bars: 200 nm. (b) Scanning probe microscopy (SPM) results of control surface (top) and EV immobilized surface (bottom). Only the EV immobilized surface exhibited vesicle‐like structures. Scanning area is 5 × 5 μm. Scanning mode is tapping mode, scanned for 512 lines with 10‐nm tip‐radius cantilever. (c) Size analyses of EM images. Sizes of detected EV‐like structures were analyzed using ImageJ software. Black bars = SEM, gray bars = SPM. *n* > 200 EV‐like structures. (d) Surface passivation test. The DDS surface passivated with 0.2% Tween‐20 showed an excellent protein repelling ability (top), whereas the surface without passivation showed a large amount of non‐specific antibody adsorption (bottom). (e) Demonstration of single‐EV imaging conditions, and (f), average EV signal counts per imaging field. Six combinations of surfaces were tested for single‐EV imaging. EVs were visualized using Alexa Fluor 488 (AF488) conjugated CD9 antibody. Only the triple positive (biotin‐BSA+/NuetrAvidin+/biotin‐EV+) condition exhibited substantial amounts of CD9 signals from immobilized EVs. Size of images: 45 × 45 μm, Bars = mean ± S.D. (*n* = 8)

To ascertain that the surface actually immobilized EVs, a surface was incubated with HEK293 EVs that had been isolated using differential ultracentrifugation (DUC‐EVs). The surface was examined using a scanning electron microscope (SEM) and a scanning probe microscope (SPM). Both images of control surfaces revealed only tiny speckles; that is, EVs were not present on the surfaces (Figure 2a,b, control surface). In contrast, the both EM images of the EV‐immobilized surfaces showed distinct EV‐like structures (Figure 2a,b, EV surface). The SEM analysis detected ∼5.3 EV‐like structures per μm (Février & Raposo, 2004) scanning area, and the footprint size of detected EVs was approximately 99 ± 46 nm (Figure 2c, **black bars**). SPM identified ∼4.3 signals EV‐like signals per μm (Février & Raposo, 2004) scanning area, and the footprint size of the signals was approximately 118 ± 41 nm (Figure 2c, **gray bars**); both numbers are similar to those obtained using SEM analysis. Both EM analyses showed similar amounts of EV‐like structures having similar size distributions, and these results agree well with previously‐known EV sizes, so we conclude that the DDS‐Tween surface successfully immobilized a significant number of EVs.

The protein‐repelling ability of the surface was then assessed by binding of Alexa Fluor 488 conjugated secondary antibodies (2 μg/ml) in the absence of EVs: if the surface can effectively repel binding of non‐specific proteins, it will show only a small number of fluorescent signals. A control surface without passivation showed a large amount of fluorescent signals from non‐specifically adsorbed antibodies (Figure 2d, DDS w/o Tween20), whereas the Tween‐passivated DDS surface exhibited almost no non‐specific adsorption of antibodies (average counts per field < 10) (Figure 2d, DDS‐Tween20).

To demonstrate whether the single‐EV imaging surface works properly, all combinations of components that are used for EV immobilization (biotin‐BSA, avidin, biotin‐EV) were tested. When biotinylated BSA was not introduced to the surface, very few of fluorescent signals were detected from the surface because it bore no anchors (Figure 2e). Even when the surface was properly coated with biotinylated BSA, fluorescence signals from CD9 antibodies were observed only when both the avidin and biotinylated EV were introduced to the surface (Figure 2e). When the numbers of signals from all six combinations were quantified, only the combination that used all three components showed substantial amounts of fluorescence signals from CD9‐labeled single EVs (Figure 2f). To confirm whether the detected CD9 signals were came from CD9‐positive EVs or from soluble CD9 molecules, we performed a detergent lysis analysis. As a result, the numbers of CD9 signals were decreased when EV samples were treated with SDS, indicating the vesicle structure of detergent‐treated EVs were disrupted (Figure S1a,b). Therefore, from this observation, we could conclude that the detected signals of CD9 staining were actually coming from surface‐immobilized EVs.

### 3.3 Characterization of single‐vesicle imaging conditions

**FIGURE 3:**
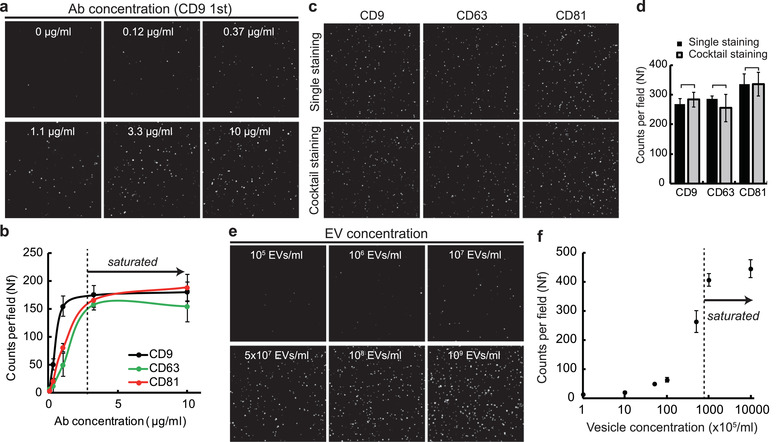
Characterization of single vesicle imaging conditions. (a,b), Characterization of antibody concentrations. The numbers of detected EV signals plateaued after 1.1 μg/ml in the case of CD9 antibody and 3.3 μg/ml in the case of CD63 and CD81 antibodies. Black line: CD9; green line: CD63; red line: CD81. Error bars = S.D. (*n* = 5). (c,d), Comparison of single staining and multiple staining of tetraspanin antibodies. The result of single and cocktail staining of CD9, CD63, and CD81 antibodies did not show significant differences in average EV signal counts. Bars = mean ± S.D. Student's t‐test, *P* > 0.1 (*n* = 8). Size of all images: 45 × 45 μm. (e,f) Characterization of EV concentration. The numbers of detected EVs plateaued at 108 particles/ml EV concentration. The linear range of detection was from ∼107 to ∼108 particles/ml. Error bars = S.D. (*n* = 8)

EV labelling and EV immobilization conditions affect the results of the single‐EV analysis, so the effects of such conditions were first characterized. First, to characterize the effect of antibody concentration, a fixed quantity of DUC‐EVs was immobilized to the prepared surfaces, then labelled with different concentrations of tetraspanin antibodies (CD9, CD63 and CD81). In the results, the EV signal counts began to saturate from 1.1 μg/ml in the case of CD9 antibody and from 3.3 μg/ml in the cases of the other two antibodies. The imaging surface appeared to effectively block the nonspecific adsorption of antibodies up to 10 μg/ml because the signals almost stopped increasing after the binding had saturated (Figure 3a,b). Because of this result, all subsequent single‐EV analyses performed in this study were conducted using an antibody concentration of 5 μg/ml.

In previous single‐molecule studies, multi‐colour analyses were often difficult due to steric hindrance (Wang et al., 2014). Therefore, whether the antibodies used in this study interfered with each other was experimentally determined. The signal counts of the cocktail labelling (labelled with all three kinds of antibodies at the same time) were not significantly different from the signal counts of single labelling (one kind of antibody for one sample); this result indicates that the tetraspanins antibodies do not interfere with each other (Figure 3c,d).

Then we characterized the effect of EV concentration on single‐vesicle analysis using DUC‐EVs. The EV signals of single‐vesicle imaging were distinguishable from the background at an EV concentration of 5 × 10^6^ particles/ml; the signals started to saturate at ∼10^8^ particles/ml (Figure 3c,d). In a few cases, the trend varied noticeably depending on batches of EV samples. From the results of further purity analysis, we determined that the purity of samples might affect the results of the single‐vesicle analysis (Figure S2a,b). For example, low‐purity EV samples contain a relatively large amount of protein contaminants that compete with EVs for binding sites on the imaging surface (Figure S2c). However, in an additional experiment we confirmed that that proportions of each tetraspanin‐positive EVs were not affected by the total EV counts of the analysis. For example, while total EV counts were changed from ∼250 to ∼80, the proportions of CD9, CD63, and CD81‐positive signals were almost unchanged (Figure S3a,b).

We also tested effect of Opti‐Prep density gradient medium for single‐EV analysis; the analysis result was not affected by addition of up to 3% Opti‐Prep (Figure S4a,b). All subsequent single‐EV analyses in this study were performed based on the conditions that were characterized here (EV concentration > 10^7^ particles/ml and Opti‐Prep concentration < 3%).

### 3.4 Comparison of different EV‐immobilization strategies

To effectively capture EVs on the surface regardless of their antigen expressions, we exploited a direct biotin‐avidin interaction. The EV‐immobilization strategy could critically affect the result of the EV analyses, so the immobilization should be demonstrated to be valid for the purpose of analysis. For example, if EVs are immobilized using surface‐tethered CD9 antibody, the result will only reflect the characteristics of CD9‐positive EVs, instead of the whole population. Therefore, using a surface‐tethered antibody strategy for studying EV diversity might provide a biased result that could distort our understanding of EV diversity. For this reason, prior to analysing the diversity of EVs, we tested experimentally whether our immobilization strategy provides unbiased analysis of EV diversity.

The DUC‐isolated EVs were immobilized on surfaces using the biotin‐avidin interaction and surface‐tethered CD81 antibody, and the surfaces were imaged using CD9, CD63, and CD81 tetraspanin antibodies (Figure 1: biotin‐avidin, Figure S5a: surface‐tethered CD81). The EV‐immobilized surfaces using biotin‐avidin interaction and surface‐tethered antibody showed clear single‐EV images in all three tetraspanin labellings (Figure S5b). When the numbers of each EV signals were quantified and compared with the WB of the same DUC‐EVs using the same set of antibodies, the biotin‐avidin sample showed the most similar tetraspanin expressions to the WB results (Figure S5c,d). The surface‐tethered CD81 immobilization showed distinctly lower counts of CD63‐positive EV signals than the counts CD9‐ or CD81‐positive EV signals. Therefore, we determined that the biotin‐avidin strategy can provide the most unbiased result that also agreed well with the results of the conventional method.

### 3.5 Characterization of tetraspanin expressions in individual EVs by co‐localization analysis

To investigate tetraspanin marker expression profiles in individual EVs, multi‐channel imaging and co‐localization analysis of the marker signals in individual EVs is essential. The reliability of the co‐localization analysis was first demonstrated using EV‐sized nanoparticles (multi‐colour, blue and red nanoparticles, all 100 nm in diameter). From the multi‐channel images of multi‐colour nanoparticles (emission: ∼400 to 700 nm), we first confirmed that the microscope setup could reliably detect all four colour channels without bias (Figure S6a,b). When the co‐localizations of the signals acquired from the different colour channels were analyzed, the signals detected from the multi‐colour nanoparticle sample showed ∼100% co‐localizations, whereas the signals detected from a blue and red nanoparticle mixture sample showed only ∼15% co‐localizations (Figure S6c,d). In addition to the nanoparticle demonstrations, DUC‐EVs labelled with CD9 primary antibody and three different coloured secondary antibodies (Alexa Fluor 488, 546, and 647) were also subjected to the multi‐colour co‐localization analysis. The results showed at least 85% of detected signals were triple‐positives (co‐localized); this result confirms that co‐localization analysis is also valid for real EV samples (Figure S6e,f).

**FIGURE 4:**
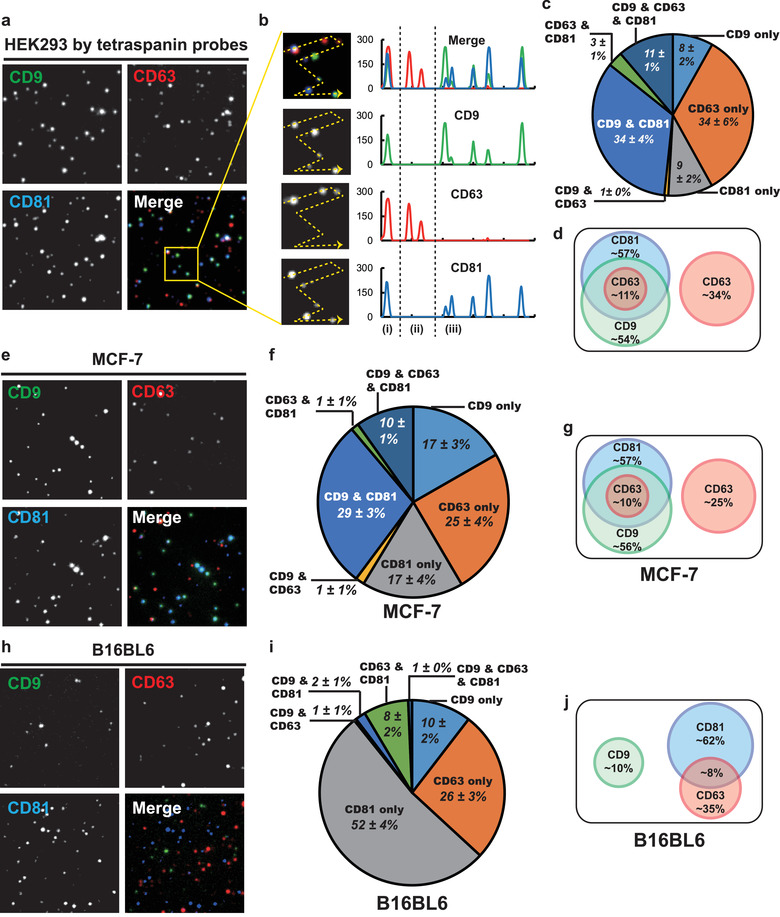
Characterization of EV populations by tetraspanin co‐localization analysis. (a), Representative single‐vesicle fluorescence images of HEK293 DUC‐EVs. The CD9, CD63, and CD81 images were coloured in green, red and blue, respectively. Size of images: 17 × 17 μm. (b) Intensity line scan of selected area (yellow box). Region (i) CD9∙CD63∙CD81 triple‐positive EV, region (ii) CD63 single‐positive EVs, and region (iii) CD9∙CD81 double‐positive EVs. Size of enlarged images: 5 × 5 μm. (c) Pie chart of EV populations expressing different combinations tetraspanins. (d) Venn diagram depicting tetraspanin relationships in individual HEK293 EVs. The EV populations were roughly divided into two distinct groups: CD63 single‐positive and CD9∙CD81 double‐positive EVs. (e‐g) Tetraspanin co‐localization analysis of MCF‐7 EVs. The population of MCF‐7 EVs were overall similar to HEK293 EVs. Size of images: 17 × 17 μm. (h‐j) Tetraspanin co‐localization analysis of B16BL6 EVs. The population of B16BL6 EVs was completely different compared to the other two cell lines; it showed higher single‐positive EV proportion than the other two cases. Numbers in pie charts: mean ± S.D. (*n* > 8). Numbers in Venn diagrams: percentages of total EVs

The DUC EV‐immobilized surfaces were then labelled with CD9, CD63, and CD81 tetraspanin antibodies having different colours, and the acquired tetraspanin signals were subjected to a multi‐colour co‐localization analysis to investigate how tetraspanins were expressed in individual EVs. All detected tetraspanin signals were analyzed for co‐localization and classified into seven EV subpopulations according to their tetraspanin expression patterns (Figure 4a,b). For example, the signals detected in a yellow‐boxed image showed three different HEK293 DIUC‐EV subpopulations: (i) CD9∙CD63∙CD81 triple‐positive EV; (ii) CD63 single‐positive EVs; and (iii) CD9∙CD81 double‐positive EVs (Figure 4b). In the result, ∼51% of the tetraspanin signals were not co‐localized (Figure 4c). Most of these single‐positive EVs were CD63 single‐positives (∼34%) (Figure 4c). As for co‐localized signals, ∼38% of the tetraspanin counts were double‐positive EVs, most of which were CD9∙CD81 double‐positive EVs (∼34%) (Figure 4c). The proportions of CD9∙CD63 and CD63∙CD81 double‐positive EV were negligible (<3%). However, CD9∙CD63∙CD81 triple‐positive EVs comprised a substantial portion (∼11%) of total EVs (Figure 4c). When all correlations among tetraspanin expressions of HEK293 DUC‐EVs were simplified as a Venn diagram, the populations were roughly divided into two groups: one mainly composed of CD9∙CD81 double‐positive EVs, and one composed of CD63 single‐positive EVs (Figure 4d).

However, EVs that are double‐positive and triple‐positive for tetraspanin could be EV aggregates, rather than individual EVs. To test this possibility, we analyzed a 0.20‐μm filtered DUC‐EV sample for tetraspanin co‐localizations. This 0.20‐μm filtration significantly decreased total detected EV counts but did not alter the tetraspanin expression profiles of individual EVs (Figure S7a‐c). Therefore, we concluded that the EV aggregates had been removed during the sample preparation process by aggregate‐cleaning centrifugation step performed after the DUC (Methods section), and that co‐localized tetraspanin signals were unlikely to be EV aggregates.

To evaluate whether the analysis is broadly applicable for different EV samples, the tetraspanin co‐localization analysis was applied to EVs that had been produced by different cell lines. The EVs were isolated using the DUC method from MCF‐7 human breast cancer and B16BL6 mouse melanoma cell cultures, and both types of EV were analyzed using the same set of tetraspanin antibodies that had been used for the HEK293 EV analysis. The analysis of DUC‐isolated MCF‐7 EV showed a similar result to that of HEK293 DUC‐EV analysis (Figure 4e,f). The MCF‐7 EVs had slightly higher proportions of CD9 and CD81 single‐positive EVs and slightly lower proportions of CD63 single‐positive EVs, but the overall profile of the tetraspanin expressions in individual EVs was almost the same as in HEK293 EVs (Figure 4d and g). In contrast, the analysis of DUC‐isolated B16BL6 EV showed a distinctively different result; only < 10% of total tetraspanin‐positive EV signals were co‐localized and most of them were single‐positive EVs (Figure 4h,i). The overall tetraspanin expression profile of the B16BL6 EVs was completely different than those of to the other two cell lines (Figure 4d, g, and j). These results suggest that the TIRF‐based single‐EV analysis can successfully detect multiple marker expressions in individual EVs from various cell types.

### 3.6 Characterization of lipid expressions in individual EVs by co‐localization analysis

Lipids are another major components of EVs, so analysis of lipid expressions in individual EVs may provide insight into the nature of EVs. Therefore, HEK293 DUC‐EVs were labelled using three different lipid probes, and the acquired lipid signals were then analyzed for co‐localizations. The three lipid specific probes were cholera toxin‐β (CTB), annexinV (AV) and Di‐dye. CTB molecules bind to ganglioside GM1, which is one of the representative components of the membrane micro‐domain, called lipid rafts (Blank et al., 2007). AV molecules have an affinity to phosphatidylserines (PS), which are mainly located at the inner leaflet of plasma membrane, so AV molecules are used to detect apoptotic cells (Van Engeland et al., 1998). Di‐dyes are lipophilic carbocyanine tracers that are used to label plasma membrane, but specific targets and labelling mechanisms are not well defined. Although these lipid‐labelling probes have different targets and mechanisms, all of them have been used for EV labelling in previous literature (Lai et al., 2015; Yost et al., 2007).

When the co‐localization of lipid probe positive signals were analyzed, all detected EVs that expressed the lipids were classified into three major subpopulations. This result indicates that the lipid expressions in DUC‐EVs were less heterogeneous than the tetraspanin expressions (Figure S8a,b). Most of the single‐positive EVs were CTB single‐positives (∼60%), whereas only ∼2% of DiI‐positive EVs and AV‐positive EVs were single‐positive (Figure S8b). Among the double‐positive EVs, only CTB∙DiI double‐positive EVs were substantially common (∼29%); but CTB∙AV and DiI∙AV double‐positive EVs were not observed (both 0%). Finally, ∼5% of EVs were positive for all three probes. When all correlations between lipid expressions were simplified as a Venn diagram, the EV populations classified by lipid expressions exhibited a distinct hierarchical structure [AV ⊂ DiI ⊂ CTB] (Figure S8c).

Further analysis of the correlation between CTB‐labelled EVs and tetraspanin‐positive EVs could confirmed that almost half of the CTB‐labelled EVs were negative for tetraspanin expression (Figure S8d). Although this population was not further investigated in this study, it was presumed to be an EV subpopulation that is enriched with GM1 lipids but does not express tetraspanins. Similarly, the single‐EV co‐localization analysis can be further used to investigate the correlation of numerous combinations of markers. However, in this study, instead of using other combinations of markers, we decided to use the three tetraspanin combinations to further investigate how different EV isolation methods affect the population of isolated EVs.

### 3.7 Conventional characterization of EV fractions acquired from different isolation methods

We then use the tetraspanin co‐localization analysis to investigate the EVs that had been isolated using three frequently‐used isolation methods: DG, buoyant DG (BDG) and size exclusion chromatography (SEC) (Figure S9). DG and BDG purify EVs according to sedimentation number (Sv) and density, and SEC purifies EVs according to size. Small impurities like proteins have small Sv, so they stay in the sample fractions in DG and BDG, but have low mobility in an SEC column, so will be eluted in the later fractions. Due to these differences in separation principles, the methods are expected to isolate different EVs. In this study, for DG and BDG, we used 0%, 10%, 20% and 30% Opti‐Prep as density‐gradient media, and acquired all fractions and subjected them to single‐EV tetraspanin analysis. When SEC was used, we tested all acquired fractions (#5 to #20) for protein amount, particle amount and tetraspanin expressions, then selected the four fractions that expressed the largest amount of tetraspanins (#8, #9, #10 #11) for further analysis (Figure S10a‐c). All fractions were obtained as 500‐μl final volume from the same amount of initial culture medium.

Before being used for the conventional and single‐EV analysis, all acquired fractions were first analyzed using nanoparticle tracking analysis (NTA) and TEM to test whether or not the fractions actually contained isolated EVs. The size distributions measured using NTA confirmed that all acquired fractions contained EV‐sized particles (Figure S11a‐c); the fractions that had heaviest density (DG/20‐30 and BDG/20‐30) showed noticeably larger particles than the other fractions (Figure S11a,b). However, the fractions acquired from SEC did not show any significant difference in size distribution; this result suggests that the SEC might not have sufficient resolution to isolate different sizes of EVs (Figure S11c). The TEM observations confirmed that all fractions included numerous EV‐like structures (Figure S11d‐f).

**FIGURE 5:**
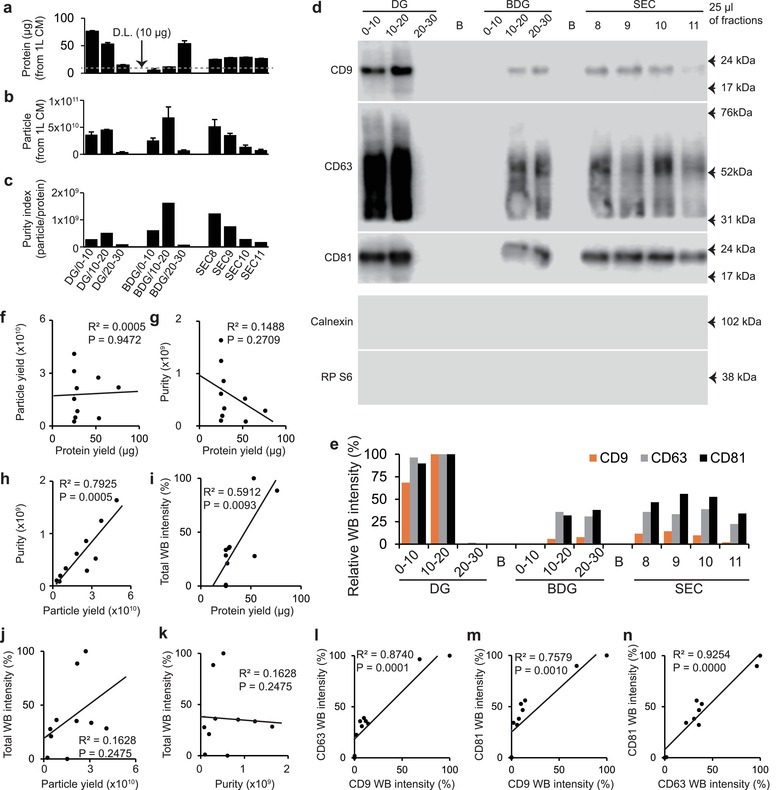
Characterization of EV fractions using conventional analyses. (a) Protein amount of EVs per 1L CM and (b), particle amount of EVs per 1L CM of all fractions. Protein amount < 10 μg could not be detected by Bradford protein assay. Bars = mean ± S.D. (*n* = 3 for protein, *n* = 6 for particle). DL, detection limit. (c) Purity index of the fractions. Purity of samples was calculated as particles per microgram protein. (d) Western blot analysis (WB) of EV fractions and (e), quantification of WB intensities. Expressions of EV‐positive markers (CD9, CD63, and CD81) and ‐negative markers (Calnexin and ribosomal protein S6) were analysed using WB. Same volumes (25 μl) of each fraction were analyzed under the same condition. Different fraction showed different tetraspanin expressions, but none of the fractions showed EV‐negative markers. (f‐k), Correlation analyses between conventional analyses and l‐m, correlation analysis between the each tetraspanin results of WB. All correlation analyses were investigated using linear regression. In all linear regressions, individual points represented mean values of each fractions. Solid lines: linear regressions; R2: determination coefficient of the regression. Regressions that have *P* < 0.05 are considered statistically significant

All acquired fractions were first analyzed using conventional EV analyses. Protein and particle yield of EVs from 1 L cultured media (CM) were quantified for each fraction, and the numbers of particles per microgram protein in each were calculated to estimate the purities. Tetraspanin WB was performed using the same 25 μl of each fraction, and the resulting bands were detected under the same detection conditions. The intensities of the bands were quantified using ImageJ software (Figure 5d,e). Protein quantifications indicated that the DG/0‐10 fraction isolated the largest number of EVs, whereas particle quantifications indicated that the BDG/10‐20 fraction isolated the largest number of EVs (Figure 5a,b). The purity index also indicated that the BDG/10‐20 fraction isolated the purest EVs (Figure 5c). However, the WB results only partially agreed with the protein quantifications; WB indicated that the DG/0‐10 and DG/10‐20 fractions isolated the largest number of EVs and that other six fractions except DG/20‐30 and BDG/0‐10 fractions showed a similar intermediate levels of tetraspanin expression. When the correlations between the conventional quantifications of all fractions were investigated, the ‘protein and WB’ pair and the ‘particle and purity’ pair showed a moderate and statistically significant correlation (R^2 ^> ∼0.6, *P* < 0.01) (Figure 5h,i). However, the protein yield did not show any correlation with either particle yield or purity index, and particle yield and purity index also did not show any correlations with WB intensity (Figure 5f,g and j,k). To summarize the results of these analyses, either the correlation among the results of the conventional analyses is low, or the different EV fractions isolated different subpopulations of EVs. In contrast, when the WB intensities of each tetraspanin results were compared, all of the pairs showed strong and statistically significant linear correlations (R^2 ^> ∼0.75, *P* < 0.01) (Figure 5l‐n). The results of analysis did not show consistent conclusions, so further analyses using single‐EV imaging and co‐localization were performed in the following sections.

### 3.8 Single‐EV tetraspanin co‐localization analysis of the EV fractions acquired from different isolation methods

**FIGURE 6:**
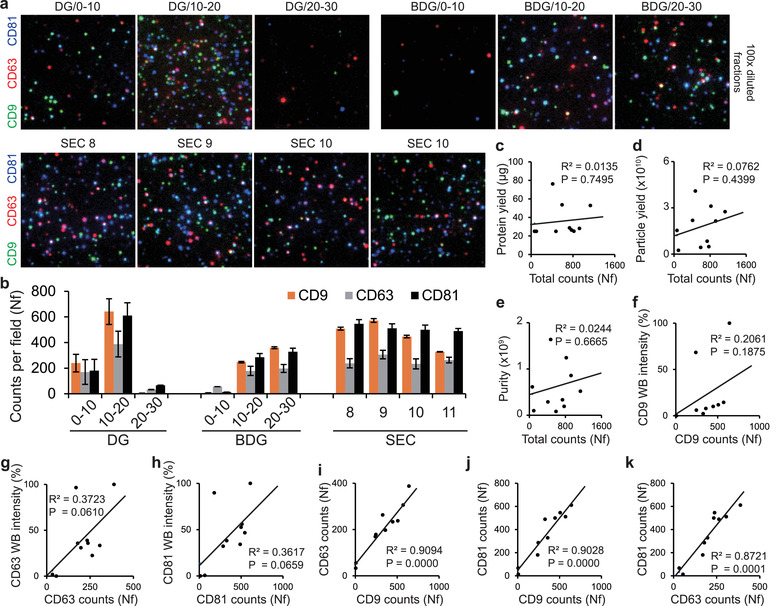
Single‐EV tetraspanin analysis of EV fractions from DG, BDG and SEC. (a), Single EV tetraspanin expression images of EV fractions. EVs expressing tetraspanin markers were analyzed using single‐EV imaging. The CD9, CD63, and CD81 signals were coloured in green, red and blue, respectively. Size of images: 17 × 17 μm. (b) Quantification of tetraspanin signals detected from EV fractions. Numbers of tetraspanin signals were quantified from the images (45 × 45 μm). Bars = mean ± S.D. (*n* = 9). (c‐h) Correlation analyses between conventional analyses and single‐EV tetraspanin analysis. The correlation between conventional analyses and single‐EV tetraspanin counts were investigated using linear regression. None of the analyses showed statistically significant correlation. (i‐k) Correlation analyses between tetraspanin counts of single‐EV analysis. The correlation between tetraspanin counts of single‐EV analysis were investigated using linear regression. All three regressions showed statistically significant correlations. In all linear regressions, individual points represented mean values of each fraction. Solid lines indicated linear regressions and R2 indicated determination coefficient of the regression. Regressions that have *P* < 0.05 are considered statistically significant

To experimentally confirm whether the isolated EV fractions contain different subpopulations or not, we analyzed the fractions using the single‐EV tetraspanin analysis. In the results, the DG/10‐20 fraction showed the highest counts for all three tetraspanins (Figure 6a,b). The SEC fractions also showed relatively high tetraspanin counts, whereas DG/20‐30 and BDG/0‐10 fraction showed low tetraspanin counts which were even lower than the counts of DUC‐EVs (Figures 4a and 6a). The DG/0‐10 and BDG/20‐30 fractions showed similar numbers of tetraspanin, which were also similar to the numbers of DUC‐EVs (Figures 4a and 6a). Before analysing co‐localizations of tetraspanin signals detected from each fraction, the correlations between the results of tetraspanin counts and conventional analyses were first investigated. As a result, the quantifications of protein, particle and tetraspanin WB did not show significant correlations with the counts from the single‐EV tetraspanin analyses (Figure 6c‐e). Even when each tetraspanin count of the fractions was then separately compared with the WB intensities, none of the pairs showed a significant correlation (*P* > 0.05) (Figure 6f‐h). However, similar to the case of the WB results, each single‐EV tetraspanin count of the fractions showed very strong and statistically significant linear correlations (R^2^ > ∼0.87 and *P* < 0.0001) (Figure 6i‐k). These results confirm that the simple tetraspanin counts of single‐EV analysis did not provide new insights, and also had little correlation with the results of conventional analyses.

**FIGURE 7:**
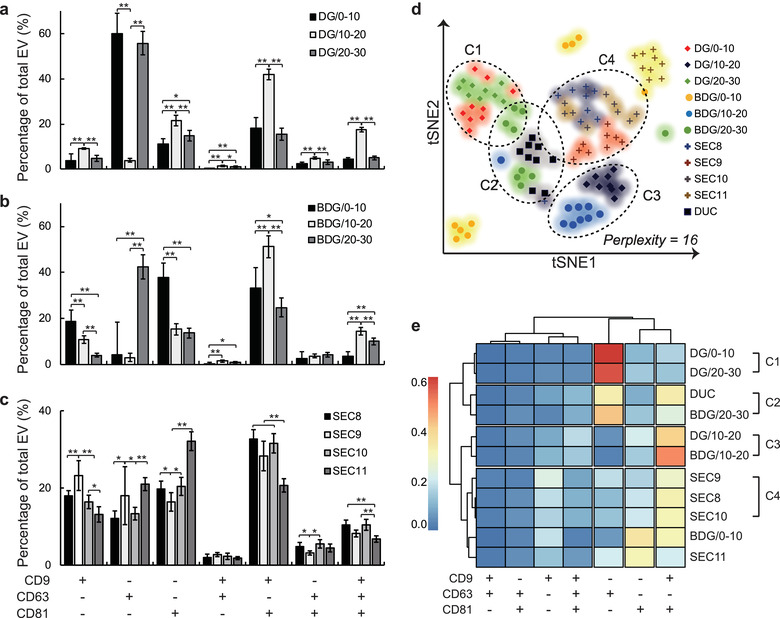
Single EV tetraspanin co‐localization analysis of all EV fractions. Tetraspanin co‐localization analysis of (a) DG fractions, (b) BDG fractions, and (c) SEC fractions. Co‐localization analysis of all fractions showed seven different EV populations by tetraspanin expression profiles. Bars = mean ± S.D., Student's t‐test, pairs showed statistical significant differences were indicated by brackets with *: *P* < 0.05, **: *P* < 0.01 (*n* > 9). (d) t‐distributed stochastic neighbour embedding (t‐SNE) visualization of all EV fractions. Seven‐dimensional population information of each fraction was reduced to two‐dimensional space by t‐SNE with perplexity value = 16. Fractions that were close to each other were grouped into clusters (c1‐c4). (*n* = 9). (e) Clustering heat map analysis of EV fractions. Average proportions of each population were expressed as coloured block (high: red, low: blue) (*n* = 9). Similarities between the EV fractions were calculated using the Euclidean distance and the similar fractions were grouped and separated by the gaps. The clusters identified in tSNE analysis are also indicated on the right side of the heat map

Therefore, tetraspanin co‐localizations analysis of each fractions were performed to further investigate the differences and similarities among the fractions. In the case of DG fractions, EV populations in DG/10‐20 fraction showed a distinctively different pattern of tetraspanin expressions compared to the other two DG fractions (Figure 7a). More than 50% of EVs isolated from DG/0‐10 and DG/20‐30 fractions were CD63 single‐positive EVs, whereas the largest population of DG/10‐20 fraction was CD9∙CD81 double‐positive EVs (Figure 7a). The BDG fractions also showed distinct differences in tetraspanin co‐localization analysis (Figure 7b). The BDG/10‐20 fraction contained mostly CD9∙CD81 double‐positive EVs, whereas the BDG/20‐30 fraction contained mostly CD63 single‐positive EVs (Figure 7b).

On the contrary, the result of SEC fractions did not show many differences among fractions: some statistically‐significant differences were observed between the fractions, but the extent was less significant than the cases of the DG or BDG fractions (Figure 7c). The SEC fractions showed comparatively evenly‐distributed populations, and the proportions of CD9, CD63, and CD81 single‐positive and CD9∙CD81 double‐positive EV populations were the four major populations (Figure 7c).

EVs in each fraction were classified into seven populations that had different tetraspanin expression profiles, it is difficult to investigate correlations among them by direct comparison. Therefore, the results were further visualized using t‐distributed stochastic neighbour embedding (t‐SNE) and clustered heat map analyses (Figure 7d,e). In both analyses, some of the fractions clustered together; that is, these fractions have similar population compositions. For example, the DG/10‐20 and BDG/10‐20 fractions were clustered together in both analyses but the DG/0‐10 fraction was not; this result suggest that the DG/10‐20 and BDG/10‐20 fractions isolated similar populations of EVs, and that they were different than the EVs in the DG/0‐10 fraction.

The heat map result showed four clusters. The first and third clusters were exactly matched with the C1 and C3 clusters of tSNE analysis; this clusters indicated that isolation methods that exploit density most effectively separated CD9∙CD81 double‐positive and CD63 single‐positive EVs, which were the two major populations of DUC‐EVs (Figures 4d and 7e). The fourth cluster and C4 cluster of tSNE showed that all SEC fractions isolated similar populations of EVs, and also showed that the SEC isolation separated different populations of EVs compared to the methods that exploit density gradients (Figure 7d,e).

Lastly, to reinvestigate the correlation between the conventional analyses and single‐EV analysis that failed in the previous section, we performed the correlation analysis again, but only with the fractions that were confirmed to have similar populations in the tetraspanin co‐localization analysis (SEC8, SEC9, SEC10, SEC11, and BDG/0‐10 fractions) (Figure 7e, **bottom five fractions**). As a consequence, the correlation between the single‐EV tetraspanin counts and tetraspanin WB became strong and statistically significant (Figure S12d‐f). However, the tetraspanin counts still did not show any correlations with protein yield, particle yield or purity (Figure S12a‐c).

## 4 DISCUSSION

Although the diversity of EVs and the presence of their subpopulations have been well demonstrate by previous studies, analytical methods that can quantitatively investigate this feature have rarely been developed. Available EV analysis methods have not been able to answer critical questions about EV biology, such as which EV subpopulations exert specific biological functions, or which subpopulations of EVs from blood provide information for disease diagnostics. Although some of the recently‐developed technologies have provided tools to investigate subpopulations of EVs, the use of such advanced technologies has often been limited. In this study, to establish the technical foundation to find answers for these important questions, we introduced a new single‐ vesicle analysis method that can visualize individual EVs and characterize their marker expressions. The method uses a TIRF microscope, which is already widely used, so various researchers can easily apply the method to their own research. For a reliable and reproducible analysis, every aspect of the analysis such as imaging surface‐EV interaction, imaging surface‐probe interaction, EV labelling conditions and accuracy of co‐localization analysis were thoroughly characterized in the manuscript. The method can be used along with conventional EV analyses because it only requires a simple biotinylation of EVs that is compatible with most of existing analysis methods. The strong EV‐anchoring property of the surface allows a reliable co‐localization analysis of multiple markers, and the effective protein‐repelling property of the surface enables rapid and efficient fluorescent labelling of EVs. With these advantages, the entire analysis, including EV labelling, took only approximately 1 h. This rapid and efficient EV analysis is a great advantage over conventional methods, which all require lengthy EV labelling and washing steps that also affect the diversity of EV samples.

From the results of the application of our single‐EV analysis to HEK293 EVs, we identified unprecedented expression patterns of tetraspanins and lipids in individual EVs. The analysis revealed two distinct subpopulations from the DUC‐isolated HEK293 EVs by tetraspanin expression pattern: one is CD9∙CD81 double‐positive EVs and the other is CD63 single‐positive EVs. Although we did not further investigate to identify the biological implications of these populations, similar observations have been reported previous literatures. A study that used flow cytometry showed co‐partitioning of CD9 and CD81 tetraspanin molecules into HIV‐1, and another showed that EVs that express CD9 and CD81 had similar sizes using size‐based EV separation technique (Dahmane et al., 2019; Jeong, Han, Cho, Gianchandani, & Park, 2018). Our analysis also showed that most of the CD63‐positive EVs were CD63 single‐positive, but once co‐localized, they were CD9∙CD63∙CD81 triple‐positives. This unique pattern was also observed in the control experiment using the surface‐tethered CD81 antibody; the number of detected CD63‐positive EVs was significantly reduced in EVs immobilized by CD81 antibody compared to EVs immobilized by biotin‐avidin interaction. However, from the analysis result of EVs isolated from the B16BL6 cell line, we also confirmed that these tetraspanin expression patterns were not always observed in all types of cells. For example, a previous literature that investigated tetraspanin expression profiles of porcine seminal plasma EVs reported that CD9 and CD63 were co‐expressed in exosomes in their flow cytometric EV analysis (Barranco et al., 2019). In addition, the co‐localization analysis result of lipid probes and tetraspanins implied possibility that there might be plenty of undetected EVs on the imaging surface. Therefore, to verify biological meanings of the identified EV subpopulations and to identify surface‐immobilized but undetected EV populations, further investigations with a wide variety of additional EV markers are required.

To demonstrate the advantages of the single‐EV analysis in a real EV applications, we performed population analysis of EV fractions acquired from three most frequently used EV isolation methods. The tetraspanin co‐localization analysis effectively showed the similarity and dissimilarity between the fractions, which could not be revealed by any of conventional analyses. For example, tetraspanin WB analysis of the BDG/10‐20 and SEC #10 fractions showed almost the same tetraspanin expression profiles, whereas single‐EV co‐localization analysis clearly illustrated their differences in tetraspanin expression profiles. The fractions acquired from different density gradient layers showed distinctively different tetraspanin expression patterns, which is indicating density‐based isolation methods were effective in separating a heterogeneous DUC‐EV sample into more refined subpopulations. On the contrary, the fractions acquired from SEC did not show much difference in tetraspanin expression profiles, and from this result, we can confirm that the SEC methods might have difficulty separating EVs that express different tetraspanins.

In summary, we demonstrated a single‐EV analysis that can visualize expressions of multiple EV‐related markers in individual EVs. By analysing the co‐localizations of tetraspanin signals, we revealed distinct EV populations expressing different combinations of tetraspanin. Although we tested only a limited number of EV markers, the analysis successfully revealed that the different isolation methods yielded different characteristics of EV fractions. Further investigations using different sets of antibodies or EVs from different sources will provide more valuable information regarding the biology of EVs. We expect that the proposed analysis method will be a powerful tool to investigate the characteristics of EVs.

## CONFLICTS OF INTEREST

The authors declare the following competing interests: J.P. is a founder of ExosomePlus, Inc. that partially supported this work.

## AUTHOR CONTRIBUTIONS

Chungmin Han and Jaesung Park designed the overall experiments. Chungmin Han, Jaehun Jung and Hyunjin Lee performed surface treatment and microfluidic channel preparations. Chungmin Han, Johan Yi, Hyunjin Lee, Yongmin Kwon, Hyejin Kang carried out EV isolation and electron microscopy. Hyejin Kang and Minsu Kang cultured cells and performed western blotting. Johan Yi and Jingeol Lee conducted nanoparticle tracking analysis. Chungmin Han established overall methods, built the TIRF microscope system, performed individual EV analysis, wrote the MATLAB script for image processing, analyzed data, prepared figures, and wrote the manuscript. Jaesung Park managed the project and helped figure preparation, and manuscript writing. The manuscript was written through contributions of all authors. All authors have given approval to the final version of the manuscript.

## Supporting information



Supporting Information

Supporting Information

Supporting Information

Supporting Information

Supporting Information

Supporting Information

Supporting Information

Supporting Information

Supporting Information

Supporting Information

Supporting Information

Supporting Information

## Data Availability

The authors confirm that the data supporting the findings of this study are available within the article and its supplementary materials.

## References

[jev212047-bib-0001] Aggarwal, V. , & Ha, T. (2016). Single‐molecule fluorescence microscopy of native macromolecular complexes. Current Opinion Structure Biology, 41, 225–232 10.1016/j.sbi.2016.09.006 27662375

[jev212047-bib-0002] Barranco, I. , Padilla, L. , Parrilla, I. , Álvarez‐Barrientos, A. , Pérez‐Patiño, C. , Peña, F. J. , Martínez, E. A. , Rodriguez‐Martínez, H. , & Roca, J. (2019).Extracellular vesicles isolated from porcine seminal plasma exhibit different tetraspanin expression profiles. Scientific Reports, 9(1), 11584. 10.1038/s41598-019-48095-3 31399634 PMC6689046

[jev212047-bib-0003] Blank, N. , Schiller, M. , Krienke, S. , Wabnitz, G. , Ho, A. D. , & Lorenz, H.‐.M. (2007). Cholera toxin binds to lipid rafts but has a limited specificity for ganglioside GM1. Immunology Cell Biology, 85, 378–382 17325693 10.1038/sj.icb.7100045

[jev212047-bib-0004] Böing, A. N. , Van Der Pol, E. , Grootemaat, A. E. , Coumans, F. A. W. , Sturk, A. , & Nieuwland, R. (2014). Single‐step isolation of extracellular vesicles by size‐exclusion chromatography. Journal of Extracellular Vesicles, 3, 1–11 10.3402/jev.v3.23430 PMC4159761 25279113

[jev212047-bib-0005] Brandenburg, B. , & Zhuang, X. (2007). Virus trafficking ‐ Learning from single‐virus tracking. Nature Reviews Microbiology, 5, 197–208 17304249 10.1038/nrmicro1615 PMC2740720

[jev212047-bib-0006] Chen, C. , Zong, S. , Wang, Z. , Lu, Ju , Zhu, D. , Zhang, Y. , & Cui, Y. (2016). Imaging and intracellular tracking of cancer‐derived exosomes using single‐molecule localization‐based super‐resolution microscope. ACS Applied Materials & Interfaces, 8, 25825–25833 27617891 10.1021/acsami.6b09442

[jev212047-bib-0007] Choi, D.‐.S. , Kim, D.‐.K. , Kim, Y.‐.K. , & Gho, Y. S. (2015). Proteomics of extracellular vesicles: exosomes and ectosomes. Mass Spectrometry Reviews, 34, 474–490 24421117 10.1002/mas.21420

[jev212047-bib-0008] Choi, U. B. , Strop, P. , Vrljic, M. , Chu, S. , Brunger, A. T. , & Weninger, K. R. (2010). Single‐molecule FRET‐derived model of the synaptotagmin 1‐SNARE fusion complex. Nature Structural & Molecular Biology, 17, 318–324 10.1038/nsmb.1763 PMC2922927 20173763

[jev212047-bib-0009] Daaboul, G. G. , Gagni, P. , Benussi, L. , Bettotti, P. , Ciani, M. , Cretich, M. , Chiari, M. , Freedman, D. S. , Ghidoni, R. , Ozkumur, A. Y. , Piotto, C. , & Prosperi, D. (2016). Digital detection of exosomes by interferometric imaging. Scientific Reports, 6, 1–10 27853258 10.1038/srep37246 PMC5112555

[jev212047-bib-0010] Dahmane, S. , Doucet, C. , Le Gall, A. , Chamontin, C. , Dosset, P. , Murcy, F. , Fernandez, L. , Salas, D. , Rubinstein, E. , Mougel, M. , Nollmann, M. , & Milhiet, P.‐.E. (2019). Nanoscale organization of tetraspanins during HIV‐1 budding by correlative dSTORM/AFM. Nanoscale, 11, 6036–6044 30869094 10.1039/c8nr07269h

[jev212047-bib-0011] Donaldson, J. (2016). Tsne: T‐Distributed Stochastic Neighbor Embedding for R (t‐SNE). R package version 0.1-3. https://CRAN.R-project.org/package=tsne

[jev212047-bib-0012] Erdbrügger, U. , Rudy, C. K. , E. Etter, M. , Dryden, K. A. , Yeager, M. , Klibanov, A. L. , & Lannigan, J. (2014). Imaging flow cytometry elucidates limitations of microparticle analysis by conventional flow cytometry. Cytometry. Part A, 85, 756–770 10.1002/cyto.a.22494 24903900

[jev212047-bib-0013] Février, B. , & Raposo, G. (2004). Exosomes: endosomal‐derived vesicles shipping extracellular messages. Current Opinion Cell Biology, 16, 415–421 10.1016/j.ceb.2004.06.003 15261674

[jev212047-bib-0014] Gardiner, C. , Ferreira, Y. J. , Dragovic, R. A. , Redman, C. W. G. , & Sargent, I. L. (2013). Extracellular vesicle sizing and enumeration by nanoparticle tracking analysis. Journal of Extracellular Vesicles, 2, (1) 10.3402/jev.v2i0.19671 PMC3760643 24009893

[jev212047-bib-0015] Guduric‐Fuchs, J. , O'connor, A. , Camp, B. , O'neill, C. L. , Medina, R. J. , & Simpson, D. A. (2012). Selective extracellular vesicle‐mediated export of an overlapping set of microRNAs from multiple cell types. BMC Genomics, 13, 357 22849433 10.1186/1471-2164-13-357 PMC3532190

[jev212047-bib-0016] Hong, B. , Cho, Ji‐H. , Kim, H. , Choi, E.‐.J. , Rho, S. , Kim, J. , Kim, J. H. , Choi, D. H. , Kim, Y. K. , Hwang, D. , & Gho, Y. (2009). Colorectal cancer cell‐derived microvesicles are enriched in cell cycle‐related mRNAs that promote proliferation of endothelial cells. BMC Genomics, 10, 1–13 19930720 10.1186/1471-2164-10-556 PMC2788585

[jev212047-bib-0017] Höög, J. L. , & Lötvall, J. (2015). Diversity of extracellular vesicles in human ejaculates revealed by cryo‐electron microscopy. Journal of Extracellular Vesicles, 4, 28680 26563734 10.3402/jev.v4.28680 PMC4643196

[jev212047-bib-0018] Hua, B. , Han, K. Y. , Zhou, R. , Kim, H. , Shi, X. , Abeysirigunawardena, S. C. , Jain, A. , Singh, D. , Aggarwal, V. , Woodson, S. A. , & Ha, T. (2014). An improved surface passivation method for single‐molecule studies. Nature Methods, 11, 1233–1236 25306544 10.1038/nmeth.3143 PMC4245390

[jev212047-bib-0019] Im, H. , Shao, H. , Park, Y. I.l , Peterson, V. M. , Castro, C. M. , Weissleder, R. , & Lee, H. (2014). Label‐free detection and molecular profiling of exosomes with a nano‐plasmonic sensor. Nature Biotechnology, 32, 490–495 10.1038/nbt.2886 PMC4356947 24752081

[jev212047-bib-0020] Jain, A. , Liu, R. , Ramani, B. , Arauz, E. , Ishitsuka, Y. , Ragunathan, K. , Park, J. , Chen, J. , Xiang, Y. K. , & Ha, T. (2011). Probing cellular protein complexes using single‐molecule pull‐down. Nature, 473, 484–488 21614075 10.1038/nature10016 PMC3103084

[jev212047-bib-0021] Jeong, H. , Han, C. , Cho, S. , Gianchandani, Y. , & Park, J. (2018). Analysis of extracellular vesicles using coffee ring. ACS Applied Materials & Interfaces, 10, 22877–22882 29911857 10.1021/acsami.8b05793

[jev212047-bib-0022] Ji, H. , Chen, M. , Greening, D. W. , He, W. , Rai, A. , Zhang, W. , & Simpson, R. J. (2014). Deep sequencing of RNA from three different extracellular vesicle (EV) subtypes released from the human LIM1863 colon cancer cell line uncovers distinct mirna‐enrichment signatures. PLoS One, 9, e110314 25330373 10.1371/journal.pone.0110314 PMC4201526

[jev212047-bib-0023] Kao, C.‐.Y. , & Papoutsakis, E. T. (2019). Extracellular vesicles: exosomes, microparticles, their parts, and their targets to enable their biomanufacturing and clinical applications. Current Opinion in Biotechnology, 60, 89–98 30851486 10.1016/j.copbio.2019.01.005

[jev212047-bib-0024] Kim, K. L. , Kim, D. , Lee, S. , Kim, S. J. , Noh, J. E. , Kim, J. H. , Chae, Y. C. , Lee, J. B. , & Ryu, S. H. (2016). Pairwise detection of site‐specific receptor phosphorylations using single‐molecule blotting. Nature Commununications, 7, 1–10 10.1038/ncomms11107 PMC4820850 27009355

[jev212047-bib-0025] Kolde, R. (2019). Pheatmap: Pretty Heatmaps. R package version 1.0.12. https://CRAN.R-project.org/package=pheatmap

[jev212047-bib-0026] Kowal, J. , Arras, G. , Colombo, M. , Jouve, M. , Morath, J. P. , Primdal‐Bengtson, B. , Dingli, F. , Loew, D. , Tkach, M. & Théry, C. (2016). Proteomic comparison defines novel markers to characterize heterogeneous populations of extracellular vesicle subtypes. Proceedings of the National Academy of Sciences of the United States of America, 113, E968–E977 26858453 10.1073/pnas.1521230113 PMC4776515

[jev212047-bib-0027] Krämer‐Albers, E.‐.M. , & Hill, A. F. (2016). Extracellular vesicles: interneural shuttles of complex messages. Current Opinion in Neurobiology, 39, 101–107 27183381 10.1016/j.conb.2016.04.016

[jev212047-bib-0028] Lai, C. P. , Kim, E. Y. , Badr, C. E. , Weissleder, R. , Mempel, T. R. , Tannous, B. A. , & Breakefield, X. O. (2015). Visualization and tracking of tumour extracellular vesicle delivery and RNA translation using multiplexed reporters. Nature Commununications, 6, 1–12. 10.1038/ncomms8029 PMC4435621 25967391

[jev212047-bib-0029] Lee, K. , Fraser, K. , Ghaddar, B. , Yang, K. , Kim, E. , Balaj, L. ,, Chiocca, E A. , Breakefield, X, O. , Lee, H. , Weissleder, R. , &^.^ Weissleder, R. (2018). Multiplexed profiling of single extracellular vesicles. ACS Nano, 12, 494–503 29286635 10.1021/acsnano.7b07060 PMC5898240

[jev212047-bib-0030] Lee, W. , Nanou, A. , Rikkert, L. , Coumans, F. A. W. , Otto, C. , Terstappen, L. W. M. M. , & Offerhaus, H. L. (2018). Label‐free prostate cancer detection by characterization of extracellular vesicles using raman spectroscopy. Analytical Chemistry, 90, 11290–11296 30157378 10.1021/acs.analchem.8b01831 PMC6170952

[jev212047-bib-0031] Llorente, A. , Skotland, T. , Sylvänne, T. , Kauhanen, D. , Róg, T. , Orłowski, A. , Vattulainen, I. , Ekroos, K. , & Sandvig, K. (2013). Molecular lipidomics of exosomes released by PC‐3 prostate cancer cells. Biochimica et Biophysica Accta, 1831, 1302–1309 10.1016/j.bbalip.2013.04.011 24046871

[jev212047-bib-0032] Margolis, L. , & Sadovsky, Y. (2019). The biology of extracellular vesicles: the known unknowns. PLoS Biology, 17, 1–12 10.1371/journal.pbio.3000363 PMC6667152 31318874

[jev212047-bib-0033] Myong, S. , Stevens, B. C. , & Ha, T. (2006). Bridging conformational dynamics and function using single‐molecule spectroscopy. Structure, 14, 633–643 16615904 10.1016/j.str.2006.02.005

[jev212047-bib-0034] Nizamudeen, Z. , Markus, R. , Lodge, R. , Parmenter, C. , Platt, M. , Chakrabarti, L. , & Sottile, V. (2018). Rapid and accurate analysis of stem cell‐derived extracellular vesicles with super resolution microscopy and live imaging. Biochimica et Biophysica Acta. Molecular Cell Research, 1865, 1891–1900 30290236 10.1016/j.bbamcr.2018.09.008 PMC6203808

[jev212047-bib-0035] Osteikoetxea, X. , Sódar, B. , Németh, A. , Szabó‐Taylor, K. , Pálóczi, K. , Vukman, K. V. , Tamási, V. , Balogh, A. , Kittel, A. , Pállinger, E. , & Buzás, E. I. (2015). Differential detergent sensitivity of extracellular vesicle subpopulations. Organic and Biomolecular Chemisty, 13, 9775–9782 10.1039/c5ob01451d 26264754

[jev212047-bib-0036] R Core Team . (2020). R: A language and environment for statistical computing. R Foundation for Statistical Computing, Vienna, Austria. https://www.R-project.org/

[jev212047-bib-0037] Raposo, G. , & Stoorvogel, W. (2013). Extracellular vesicles: exosomes, microvesicles, and friends. Journal of Cell Biology, 200, 373–383 10.1083/jcb.201211138 PMC3575529 23420871

[jev212047-bib-0038] Roy, R. , Hohng, S. , & Ha, T. (2008). A practical guide to single‐molecule FRET. Nature Methods, 5, 507–516 18511918 10.1038/nmeth.1208 PMC3769523

[jev212047-bib-0039] Shu, S. L.a , Yang, Y. , Allen, C. L. , Hurley, E. , Tung, K. H. , Minderman, H. , Wu, Y., & Ernstoff, M. S. (2020). Purity and yield of melanoma exosomes are dependent on isolation method. Journal of Extracellular Vesicles, 1692401, 9 31807236 10.1080/20013078.2019.1692401 PMC6882439

[jev212047-bib-0040] Simpson, R. J. , Jensen, S. S. , & Lim, J. W. E. (2008). Proteomic profiling of exosomes: current perspectives. Proteomics, 8, 4083–4099 18780348 10.1002/pmic.200800109

[jev212047-bib-0041] Sitar, S. , Kejžar, A. , Pahovnik, D. , Kogej, K. , Tušek‐Žnidarič, M. , Lenassi, M. , & Žagar, E. (2015). Size characterization and quantification of exosomes by asymmetrical‐flow field‐flow fractionation. Analytical Chemistry, 87, 9225–9233 26291637 10.1021/acs.analchem.5b01636

[jev212047-bib-0042] Skliar, M. , & Chernyshev, V. S. (2019) Imaging of extracellular vesicles by atomic force microscopy. Journal of Visualized Experiments : JoVE, 1–13. 10.3791/59254 31566613

[jev212047-bib-0043] Skotland, T. , Hessvik, N. P. , Sandvig, K. , & Llorente, A. (2019). Exosomal lipid composition and the role of ether lipids and phosphoinositides in exosome biology. Journal of Lipid Research, 60, 9–18 30076207 10.1194/jlr.R084343 PMC6314266

[jev212047-bib-0044] Tauro, B. J. , Greening, D. W. , Mathias, R. A. , Ji, H. , Mathivanan, S. , Scott, A. M. , & Simpson, R. J. (2012). Comparison of ultracentrifugation, density gradient separation, and immunoaffinity capture methods for isolating human colon cancer cell line LIM1863‐derived exosomes. Methods, 56, 293–304 22285593 10.1016/j.ymeth.2012.01.002

[jev212047-bib-0045] Théry, C. , Amigorena, S. , Raposo, G. , & Clayton, A. (2006). Isolation and characterization of exosomes from cell culture supernatants and biological fluids. Current Protocols in Cell Biology, 30(1), 3–22 10.1002/0471143030.cb0322s30 18228490

[jev212047-bib-0046] Théry, C. , Witwer, K. W. , Aikawa, E. , Alcaraz, M. J. , Anderson, J. D. , Andriantsitohaina, R. , Antoniou, A. , Arab, T. , Archer, F. , Atkin‐Smith, G. K. , Ayre, D. C. , Bach, J. M. , & Zuba‐Surma, E. K. (2018). Minimal information for studies of extracellular vesicles 2018 (MISEV2018): a position statement of the International Society for Extracellular Vesicles and update of the MISEV2014 guidelines. Journal of Extracellular Vesicles, 7, 1535750 30637094 10.1080/20013078.2018.1535750 PMC6322352

[jev212047-bib-0047] Théry, C. , Zitvogel, L. , & Amigorena, S. (2002). Exosomes: composition, biogenesis and function. Nature Reviews Immunology, 2, 569–579 10.1038/nri855 12154376

[jev212047-bib-0048] Ulbrich, M. H. , & Isacoff, E. Y. (2008) Rules of engagement for NMDA receptor subunits, 105, 14163–14168. https://www.pnas.org/content/105/37/14163 10.1073/pnas.0802075105 PMC2544595 18779583

[jev212047-bib-0049] Van Der Pol, E. , Van Gemert, M. J. C. , Sturk, A. , Nieuwland, R. , & Van Leeuwen, T. G. (2012). Single vs. swarm detection of microparticles and exosomes by flow cytometry. Journal of Thrombosis Haemostasis, 10, 919–930 22394434 10.1111/j.1538-7836.2012.04683.x

[jev212047-bib-0050] Van Engeland, M. , Nieland, L. J. W. , Ramaekers, F. C. S. , Schutte, B. , & Reutelingsperger, C. P. M. (1998). Annexin V‐affinity assay: a review on an apoptosis detection system based on phosphatidylserine exposure. Cytometry, 31, 1–9 9450519 10.1002/(sici)1097-0320(19980101)31:1<1::aid-cyto1>3.0.co;2-r

[jev212047-bib-0051] Wang, Y. , Liu, Y. , Deberg, H. A. , Nomura, T. , Hoffman, M. T. , Rohde, P. R. , Schulten, K. , Martinac, B. , & Selvin, P. R. (2014). Single molecule FRET reveals pore size and opening mechanism of a mechano‐sensitive ion channel. Elife, 3, 1–21 10.7554/eLife.01834 PMC3925968 24550255

[jev212047-bib-0052] Wubbolts, R. , Leckie, R. S. , Veenhuizen, P. T. M. , Schwarzmann, G. , Möbius, W. , Hoernschemeyer, J. , Slot, J. W. , Geuze, H. J. , & Stoorvogel, W. (2003). Proteomic and biochemical analyses of human B cell‐derived exosomes: potential implications for their function and multivesicular body formation. Journal of Biological Chemistry, 278, 10963–10972 10.1074/jbc.M207550200 12519789

[jev212047-bib-0053] Yost, E. A. , Mervine, S. M. , Sabo, J. L. , Hynes, T. R. , & Berlot, C. H. (2007). Live cell analysis of G protein β5 complex formation, function, and targeting. Molecular Pharmacology, 72, 812–825 17596375 10.1124/mol.107.038075

[jev212047-bib-0054] Yuana, Y. , Koning, R. I. , Kuil, M. E. , Rensen, P. C. N. , Koster, A. J. , Bertina, R. M. , & Osanto, S. (2013). Cryo‐electron microscopy of extracellular vesicles in fresh plasma. Journal of Extracellular Vesicles, 2, 21494 10.3402/jev.v2i0.21494 PMC3895263 24455109

[jev212047-bib-0055] Zhang, H. , Freitas, D. , Kim, H. S. , Fabijanic, K. , Li, Z. , Chen, H. , Mark, M. T. , Molina, H. , Martin, A. B. , Bojmar, L. , Fang, J. , & Lyden, D. (2018). Identification of distinct nanoparticles and subsets of extracellular vesicles by asymmetric flow field‐flow fractionation. Nature Cell Biology, 20, 332–343 29459780 10.1038/s41556-018-0040-4 PMC5931706

